# DNA methylation changes that precede onset of dysplasia in advanced sessile serrated adenomas

**DOI:** 10.1186/s13148-019-0691-4

**Published:** 2019-06-14

**Authors:** Cheng Liu, Lochlan J. Fennell, Mark L. Bettington, Neal I. Walker, Joel Dwine, Barbara A. Leggett, Vicki L. J. Whitehall

**Affiliations:** 1 The Conjoint Gastroenterology Laboratory, QIMR Berghofer Medical Research Institute, 300 Herston Road, Herston, Brisbane, QLD 4006 Australia; 2 Faculty of Medicine, University of Queensland, Brisbane, QLD Australia; 3 Envoi Specialist Pathologists, Brisbane, QLD Australia; 4 The Royal Brisbane and Women’s Hospital, Brisbane, QLD Australia; 5 Department of Chemical Pathology, Pathology Queensland, Brisbane, QLD Australia

**Keywords:** Sessile serrated adenoma, Dysplasia, Methylation, Colorectal cancer, Methylation array, *MLH1*

## Abstract

**Background:**

Sessile serrated adenomas (SSAs) are common polyps which give rise to 20–30% of colorectal cancer (CRC). SSAs display clinicopathologic features which present challenges in surveillance, including overrepresentation in young patients, proclivity for the proximal colon and rarity of histologic dysplasia (referred to then as SSAs with dysplasia, SSADs). Once dysplasia develops, there is rapid progression to CRC, even at a small size. There is therefore a clinical need to separate the “advanced” SSAs at high risk of progression to SSAD and cancer from ordinary SSAs. Since SSAs are known to accumulate methylation over time prior to the development of dysplasia, SSAD backgrounds (the remnant SSA present within an SSAD) likely harbour additional methylation events compared with ordinary SSAs. We therefore performed MethyLight and comprehensive methylation array (Illumina MethylationEPIC) on 40 SSAD backgrounds and 40 matched ordinary SSAs, and compared the methylation results with CRC methylation, CRC expression and immunohistochemical data.

**Results:**

SSAD backgrounds demonstrated significant hypermethylation of CpG islands compared with ordinary SSAs, and the proportion of hypermethylated probes decreased progressively in the shore, shelf and open sea regions. Hypomethylation occurred in concert with hypermethylation, which showed a reverse pattern, increasing progressively away from the island regions. These methylation changes were also identified in *BRAF*-mutant hypermethylated CRCs. When compared with CRC expression data, *SV2B*, *MLH1*/*EPM2AIP1*, *C16orf62*, *RCOR3*, *BAIAP3*, *OGDHL*, *HDHD3* and *ATP1B2* demonstrated both promoter hypermethylation and decreased expression. Although SSAD backgrounds were histologically indistinguishable from ordinary SSAs, *MLH1* methylation was detectable via MethyLight in 62.9% of SSAD backgrounds, and focal immunohistochemical MLH1 loss was seen in 52.5% of SSAD backgrounds.

**Conclusions:**

Significant hyper- and hypomethylation events occur during SSA progression well before the development of histologically identifiable changes. Methylation is a heterogeneous process within individual SSAs, as typified by *MLH1*, where both *MLH1* methylation and focal immunohistochemical MLH1 loss can be seen in the absence of dysplasia. This heterogeneity is likely a generalised phenomenon and should be taken into account in future methylation-based studies and the development of clinical methylation panels.

**Electronic supplementary material:**

The online version of this article (10.1186/s13148-019-0691-4) contains supplementary material, which is available to authorized users.

## Background

Colorectal cancer (CRC) is the third most common cancer worldwide, responsible for approximately 860,000 deaths in 2018 [1]. CRC arises from two precursor pathways. The conventional pathway, responsible for 70–80% of CRC, follows the adenoma-carcinoma sequence and is initiated by conventional adenomas [2]. These adenomas progress slowly and increase in size and dysplasia over time, with the risk of CRC development being directly related to these parameters [3]. This provides an extended period for colonoscopic detection.

The serrated pathway is responsible for the remaining 20–30% of cases [4] and is initiated by sessile serrated adenomas (SSAs). SSAs differ from conventional adenomas in several respects. They arise at any age and are overrepresented in young patients [5, 6]. Histologically, they vary little in appearance for the majority of their dwell time prior to the development of dysplasia. However, once dysplasia develops (referred to then as SSA with dysplasia, SSAD), they rapidly progress to CRC [7]. SSADs are thought to be responsible for many “interval” CRCs which arise within the colonoscopy surveillance interval [8].

These characteristics present special challenges in surveillance. The criteria used in conventional adenomas are suboptimal, as SSAs do not increase in size significantly with age [5], and the SSAs which progress to dysplasia do so at a small size (less than 10 mm) [7, 9]. SSADs are rare, vary in morphology, are readily mistaken for other entities and progress too rapidly to be useful as a risk marker [7, 10]. On the other hand, because SSAs comprise 20% of resected colorectal polyps [11] and the majority do not develop dysplasia, treating all lesions as potentially malignant will require following up an unacceptably large number of patients.

Ideally high-risk SSAs should be identified prior to the development of dysplasia, but this is not possible based on histologic appearance alone. From a molecular aspect, SSAs are initiated by *BRAF* mutation and accumulate methylation at CpG sites over time. This accumulation is not random and can be quantified by assessing the methylation status of preselected genes. When these genes are methylated, it is referred to as the CpG island methylator phenotype (CIMP). CIMP is strongly associated with *BRAF* mutation in CRCs [12] and is almost universal in SSADs [7], while its incidence in SSAs is much more variable and correlates with increased patient age [13]. Methylation in SSAs occurs over many years, eventually reaching a threshold where critical tumour-suppressor genes are silenced by promoter methylation [14]. *MLH1* is the best characterised of these tumour-suppressor genes, and loss of MLH1 protein function results in microsatellite instability and further accumulation of mutations [15]. Histologically, detectable dysplasia then develops, and the SSA becomes an SSAD.

From this model, the residual non-dysplastic SSA in an SSAD (hereby referred to as “SSAD background”) represents the most “advanced” SSA prior to development of dysplasia and should harbour similarly advanced molecular alterations, despite being histologically indistinguishable from an ordinary SSA. *BRAF* mutation and CIMP are not useful in this context as they are present in the majority of ordinary SSAs, while *MLH1* silencing occurs too late in progression and is essentially restricted to SSADs. Identification of significant methylation events which accompany CIMP, but precede *MLH1* silencing, may translate into clinical markers which allow for identification of high-risk SSAs. We therefore analysed a large series of residual non-dysplastic SSA in an SSAD (i.e. SSAD backgrounds) and ordinary SSAs using the Infinium MethylationEPIC platform, a methylation microarray which interrogates 866,836 CpG sites across the genome.

## Results

### Patient demographics

As the SSAD backgrounds and SSAs were matched, the demographics were essentially identical (Table 1). For SSAD backgrounds, the mean age was 75.1 years, 30 were female and 36 were proximal. For SSAs, the mean age was 75.0 years, 30 were female and 39 were proximal.

**Table 1.** Patient demographics

		SSAD background (*N* = 40)	SSA (*N* = 40)	*P* value
Age (mean ± SD)		75.1 ± 8.3	75.0 ± 7.9	N.S.
Gender				
	Female	30	30	N.S.
	Male	10	10	
Colonic site				
	Proximal	36	39	N.S.*
	Distal	1	1	
	Unknown	3	0	

*Excluding SSAD backgrounds of unknown site. *SSAD* sessile serrated adenoma with dysplasia, *SSA* sessile serrated adenoma, *N.S.* not significant

### Methylation of CIMP genes

The methylation status of the five CIMP genes (*NEUROG1*, *SOCS1*, *CACNA1G*, *IGF2* and *RUNX3*) [16] was examined by both MethyLight and the MethylationEPIC array.

With MethyLight, all 35 SSAD backgrounds with sufficient DNA were CIMP-high. As part of the selection criteria, all 40 SSAs were also CIMP-high. Specifically, *NEUROG1*, *SOCS1*, *CACNA1G*, *IGF2* and *RUNX3* were hypermethylated in 35 of 35 (100.0%), 28 of 35 (80.0%), 35 of 35 (100.0%), 35 of 35 (100.0%) and 35 of 35 (100.0%) SSAD backgrounds, respectively; they were hypermethylated in 39 of 40 (97.5%), 20 of 40 (50.0%), 35 of 40 (87.5%), 37 of 40 (92.5%) and 35 of 40 (87.5%) SSAs, respectively. Only *SOCS1* methylation was significantly different between the two groups (*P* < 0.01). These results are summarised in Additional file 1: Supplementary Table 1.

With the MethylationEPIC array, for *NEUROG1*, 16 of 20 promoter-associated probes were hypermethylated in SSAD backgrounds compared with SSAs, and one of these differences was significant (*P* < 0.05, Additional file 2: Supplementary Figure 1A). For *SOCS1*, 13 of 19 probes were hypermethylated, but none of the differences were significant (Additional file 2: Supplementary Figure 1B). For *CACNA1G*, 19 of 23 probes were hypermethylated, but none of the differences were significant (Additional file 2: Supplementary Figure 1C). For *IGF2*, 9 of 20 probes were hypermethylated, but none of the differences were significant (Additional file 2: Supplementary Figure 1D). For *RUNX3*, 18 of 20 probes were hypermethylated, and five of these differences were significant (*P* < 0.05, Additional file 2: Supplementary Figure 1E).

### Methylation and expression of *MLH1*

With MethyLight, *MLH1* was methylated in 22 of 35 (62.9%) SSAD backgrounds with sufficient DNA, compared with 0 of 40 (0.0%) SSAs (Additional file 1: Supplementary Table 1). Although this difference was statistically significant (*P* < 0.01), it was likely due to the selection criterion which required all SSAs to be *MLH1* unmethylated. With the MethylationEPIC array, 19 of 24 *MLH1* promoter-associated probes were hypermethylated in SSAD backgrounds compared with SSAs, and two of these differences were significant (*P* < 0.05, Additional file 2: Supplementary Figure 1F). Of note, all 11 island-associated probes were hypermethylated, which included the two significantly different probes.

**Fig. 1.**
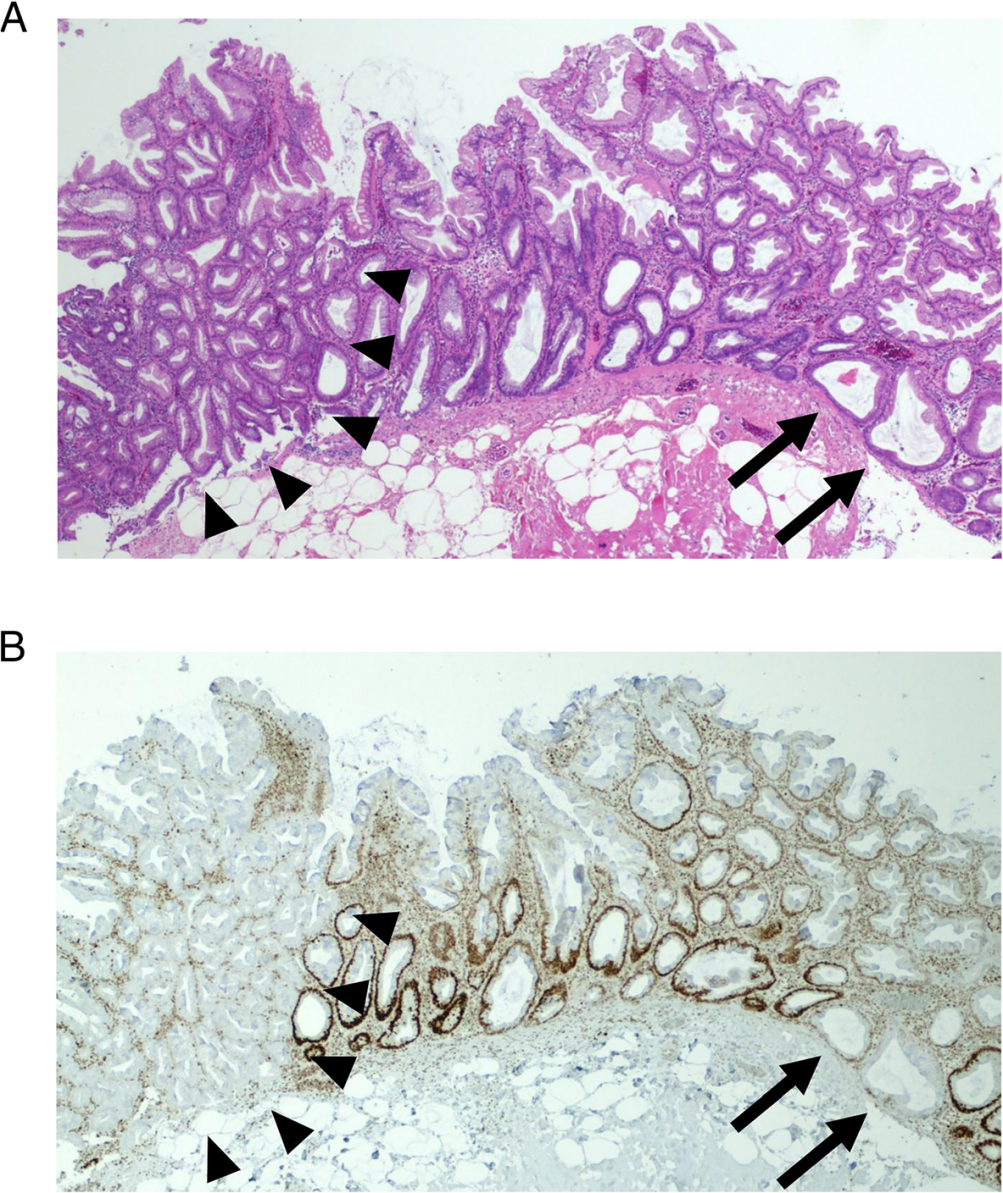
Focal MLH1 loss in the SSAD backgrounds. **a** Haematoxylin and eosin-stained section of an SSAD, with the SSAD background on the right (crypts of interest indicated by arrows) and the dysplastic portion on the left (arrowheads). **b** MLH1 immunohistochemistry for the same SSAD. There is loss of nuclear MLH1 staining in a proportion of crypts in the SSAD background (arrows), while the dysplastic portion shows complete loss of staining (arrowheads). SSAD, sessile serrated adenoma with dysplasia; SSA, sessile serrated adenoma.

Immunohistochemical MLH1 loss in isolated crypts was identified in 21 of 40 (52.5%) SSAD backgrounds (example shown in Fig. 1). These crypts were focal (less than 5% of all crypts in a given SSAD background) and not associated with any appreciable H&E differences compared with adjacent crypts with retained MLH1. Of the subset of SSAD backgrounds with sufficient DNA for MethyLight, 19 of 35 (54.3%) showed focal MLH1 loss. Fourteen of 19 (73.7%) SSAD backgrounds with MLH1 loss were *MLH1* methylated, compared with 8 of 16 (50.0%) SSAD backgrounds with retained MLH1. This difference was not significant (Additional file 3: Supplementary Table 2). As per convention, this analysis used a percentage of methylated reference (PMR) of 10% as the minimal cut off for significant methylation [16]. However, because MLH1 loss involved only a small number of crypts, whole-lesion *MLH1* methylation was unlikely to reach this threshold. We thus relaxed the cut off and examined cases with any detectable *MLH1* methylation (i.e. PMR > 0%). In SSAD backgrounds with MLH1 loss, 17 of 19 (89.5%) showed detectable *MLH1* methylation, compared with 10 of 16 (62.5%) SSAD backgrounds with retained MLH1. This difference was also not significant (Additional file 3: Supplementary Table 2).

### Global methylation differences between SSAD backgrounds and SSAs

**Fig. 2.**
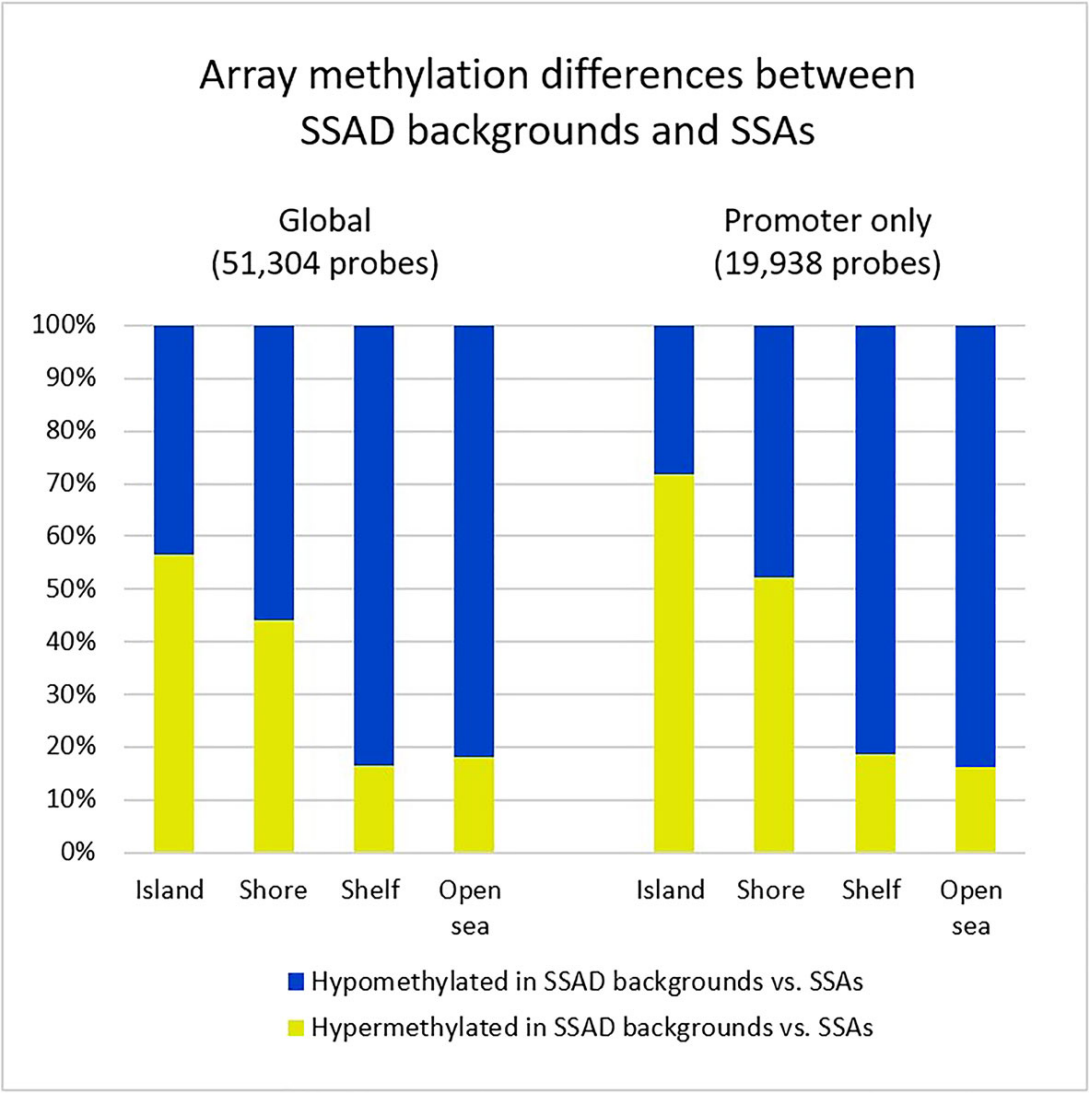
Array methylation differences between SSAD backgrounds and SSAs. Globally SSAD backgrounds are more methylated than SSAs in island regions, but the proportion of hypermethylated probes decreases towards shore, shelf and open sea regions. The same trend is accentuated when only promoter probes are analysed. SSAD, sessile serrated adenoma with dysplasia; SSA, sessile serrated adenoma.

Overall, 740,033 of 866,836 (85.4%) probes on the MethylationEPIC array met the quality control and filtering threshold and were included in the analyses. Using an adjusted *P* < 0.05, 51,304 of these probes were significantly differentially methylated between SSAD backgrounds and SSAs. The majority of the probes showed hypomethylation, rather than hypermethylation, in SSAD backgrounds (36,744 of 51,304, 71.6%). To further investigate this finding, we divided the probes into island, shore, shelf and open sea groups. With this division, SSAD backgrounds were more hypermethylated in the islands (4274 of 7561, 56.5%). However, as one moved away from the islands, the proportion of hypermethylated probes in SSAD backgrounds decreased progressively from 4195 of 9504 (44.1%) shore probes to 676 of 4097 (16.5%) shelf probes and to 5415 of 30,142 (18.0%) open sea probes. These data are summarised in Fig. 2.

### Promoter methylation differences between SSAD backgrounds and SSAs

Due to the known role of promoter methylation in SSA progression, we then limited analyses to promoter-associated probes, defined as within 1500 bp upstream of the transcription start site of a gene [17]. A total of 19,938 probes were significantly differentially methylated between SSAD backgrounds and SSAs. On dividing the probes into island, shore, shelf and open sea groups, SSAD backgrounds were more hypermethylated in the islands (3047 of 4248, 71.7%). However, as one moved away from the islands, the proportion of hypermethylated probes in SSAD backgrounds decreased progressively from 2809 of 5384 (52.2%) shore probes to 180 of 961 (18.7%) shelf probes and to 1516 of 9345 (16.2%) open sea probes. These data are summarised in Fig. 2.

### Comparison with methylation in *BRAF*-mutant CIMP-high microsatellite unstable CRC

Biologically significant methylation differences which occur from SSA to SSAD background should persist into the resultant CRC; that is, hypermethylated probes in the SSAD background should remain hypermethylated in the CRC, and similarly for hypomethylated probes. Because all of our SSADs showed loss of MLH1 staining on immunohistochemistry in the dysplastic component, they were expected to give rise to *BRAF*-mutant CIMP-high microsatellite unstable CRCs if allowed to progress. We had previously performed methylation array on 21 formalin-fixed, paraffin-embedded CRCs, using the Illumina HumanMethylation450 platform (Illumina, San Diego, USA) [18]. Six of these were *BRAF*-mutant CIMP-high microsatellite unstable CRCs, and they were compared with the 40 SSAs in the current study.

Of the 51,304 probes which showed a significant difference between SSAD backgrounds and SSAs on the MethylationEPIC platform, 27,748 were also present in the HumanMethylation450 platform. They represented 9630 hypermethylated probes and 18,118 hypomethylated probes in the SSAD background versus SSA comparison, and comparison between CRC and SSA was limited to these probes. For the SSAD background hypermethylated probes, 7173 (74.5%) were also hypermethylated in CRC, 138 (1.4%) were hypomethylated in CRC and 2319 (24.1%) were not significantly different. For the SSAD background hypomethylated probes, 10,476 (57.8%) were also hypomethylated in CRC, 874 (4.8%) were hypermethylated in CRC and 6768 (37.4%) were not significantly different. Considering only probes which showed a significant difference in the CRC versus SSA comparison, 17,649 of 18,661 (94.6%) were concordant between SSAD background and CRC, and only 1012 of 18,661 (5.4%) were discordant. The great majority of methylation changes identified in SSAD backgrounds were therefore also present in *BRAF*-mutant CIMP-high microsatellite unstable CRC.

### Identification of potential genes involved in SSA progression

To identify candidate tumour-suppressor genes silenced in the progression of SSA to SSAD background, we searched for hypermethylated islands within promoters of protein-coding genes. This identified 2936 probes, corresponding to 2290 unique protein-coding genes. The 20 most hypermethylated (highest logFC) and 20 most significantly different (lowest adjusted *P* value) probes and their corresponding genes are shown in Table 2. The complete list of all significant probes is given in Additional file 4: Supplementary Table 3.

**Table 2.** Hypermethylated promoter island probes showing largest differences between SSAD backgrounds and SSAs

Probe	Gene	Methylation logFC (SSAD background vs. SSA)*	Adjusted *P* value
Most hypermethylated probes			
cg21016956	*NOL4*	1.493751	0.000787
cg16916433	*ADAMTS19*	1.392486	0.002431
cg07733457	*ZNF594*	1.363048	0.002957
cg20388206	*NOL4*	1.290463	0.002242
cg08624472	*ARPC1B*	1.253215	0.040197
cg08326075	*SV2B*	1.251781	0.003033
cg16552945	*ARHGDIG*	1.233535	0.007083
cg14933485	*PGR*	1.200440	0.000873
cg06520273	*CCDC39*	1.198272	0.004478
cg07352001	*ZNF32*	1.197676	0.010383
cg24713878	*P2RX5*	1.195933	0.037569
cg27331401	*MLH1*/*EPM2AIP1*	1.194737	0.022763
cg15353810	*C16orf62*	1.190115	0.005143
cg17210604	*HIC1*	1.180987	0.031011
cg11224603	*MLH1*/*EPM2AIP1*	1.163986	0.004474
cg22308600	*NOL4*	1.163516	0.005631
cg22715021	*CLPSL2*	1.157144	0.035127
cg27586588	*MLH1*/*EPM2AIP1*	1.154898	0.048775
cg12609243	*CACNA2D3*	1.148465	0.002208
cg11555122	*ZNF594*	1.142647	0.005650
Most statistically significant probes			
cg16709874	*RCOR3*	1.017873	0.000382
cg21016956	*NOL4*	1.493751	0.000787
cg14933485	*PGR*	1.200440	0.000873
cg19809077	*GTF2H4*/*VARS2*	0.751390	0.000897
cg23546619	*BCAS3*	0.717448	0.001064
cg17360299	*MYCN*	0.783056	0.001130
cg06925115	*ATP2B2*	0.718108	0.001319
cg26691477	*EN1*	0.714066	0.001326
cg05116343	*BAIAP3*	0.902199	0.001433
cg24727182	*ETNPPL*	0.748564	0.001561
cg02357389	*CCDC59*/*METTL25*	0.958562	0.001634
cg09647147	*OGDHL*	0.959377	0.001654
cg07774938	*CCDC180*	0.880026	0.001682
cg11680300	*AUTS2*	1.135098	0.001693
cg02945056	*GCC1*	0.831327	0.001795
cg00142257	*LHX6*	0.789183	0.001795
cg04987474	*HDHD3*	0.561859	0.001948
cg21267231	*DUOX2*	0.557276	0.002008
cg09982069	*XPO4*	0.909124	0.002033
cg10043101	*ATP1B2*	0.940714	0.002133

*A methylation logFC value of > 0 indicates hypermethylation in SSAD background compared with SSA. *SSAD* sessile serrated adenoma with dysplasia, *SSA* sessile serrated adenoma

Because promoter methylation does not always correlate with decreased gene expression [19, 20], we then compared our list of 2290 candidate genes with publicly available CRC expression data (Fennell et al. [21], ArrayExpress E-MTAB-7036). When 18 *BRAF*-mutant CIMP-high microsatellite unstable CRCs (i.e. the CRCs expected to arise from MLH1-deficient SSADs) were compared with 32 normal mucosa samples, 870 of the 2290 protein-coding genes were differentially expressed, with 341 (39.2%) showing decreased expression. Of the top hypermethylated probes in Table 2, eight probes were associated with decreased gene expression in CRC, corresponding to *SV2B*, *MLH1*/*EPM2AIP1*, *C16orf62*, *RCOR3*, *BAIAP3*, *OGDHL*, *HDHD3* and *ATP1B2*. The complete list of all differentially expressed genes is given in Additional file 5: Supplementary Table 4.

## Discussion

Based on endoscopic and pathologic studies in the past decade, the natural history of SSAs is becoming increasingly clear. Once initiated by a *BRAF* mutation, SSAs accumulate methylation at a gradual rate over decades [13]. Despite the increase in methylation, there is little change in size and histologic appearance. After methylation reaches a certain threshold, tumour-suppressor genes such as *MLH1* are silenced by promoter methylation and histologic dysplasia, or an SSAD, develops [4]. SSADs progress rapidly to CRC which, based on microsatellite instability status, can have a superior prognosis or a poor prognosis [22].

These features present special challenges in colonoscopic surveillance, since criteria used to identify high-risk conventional adenomas are poorly applicable in SSAs. SSAs do not increase significantly in size over time [5], and dysplasia progresses too rapidly to be useful as a marker [7]. Methylation testing is a promising surrogate marker due to its long period of accumulation, and applicability is supported by the observation that virtually all SSADs are hypermethylated [7]. However, past age 60 more than 70% of ordinary SSAs are also hypermethylated [13], and the great majority of these do not develop dysplasia; the indiscriminate use of methylation panels in unselected SSAs is therefore impractical.

The non-dysplastic SSA component in an SSAD, or SSAD background as referred to in this study, represents the most “advanced” SSA possible prior to development of dysplasia. Although morphologically identical, SSAD backgrounds should differ in methylation from ordinary SSAs. To clarify these differences, we examined two homogeneous groups most likely to harbour biologically significant methylation differences. The SSAD backgrounds were all from MLH1-deficient SSADs. The SSAs were age-, gender- and site-matched to the SSAD backgrounds, with the additional requirements of also being CIMP-high and *MLH1* unmethylated via MethyLight. The CIMP-high requirement ensured these SSAs were similar to the SSAD backgrounds (which were all CIMP-high in samples with sufficient DNA for MethyLight), while the *MLH1* unmethylated requirement ensured the SSAs did not represent undersampled SSADs.

With these stringent clinical, histologic and methylation criteria, the MethylationEPIC array still identified a large number of significantly differentially methylated probes between SSAD backgrounds and SSAs (Fig. 2). Globally, although only 28.4% of probes were hypermethylated in SSAD backgrounds, they were concentrated within CpG islands, where 56.5% of probes were hypermethylated. The degree of methylation decreased as one moved away from the islands to the shore, shelf and sea regions. Due to the known importance of promoter hypermethylation-induced gene silencing in SSA progression, we then restricted analysis to the promoter regions. The trend was accentuated, where 71.7% of island-associated probes were hypermethylated, with the same decrease as one moved away from the islands. This concentration of hypermethylation in promoter-associated regions, and a tendency towards hypomethylation elsewhere, is also identified in studies comparing unselected CRC with normal mucosa [20, 23–25].

In contrast, there are few comparable studies of SSAs. Most previous methylation studies of SSAs have focused on a single gene or a panel of preselected genes [26–35]. Two array-based studies and one sequencing study have compared SSAs to normal mucosa. Dehghanizadeh et al. [36] used the HumanMethylation450 array to identify methylated genes in SSAs compared with normal mucosa, followed by RNA sequencing. Three genes were methylated and downregulated in SSAs (*BMP3*, *EPB41L3* and *CBS*). Using a customised methylation microarray, Inoue et al. [37] identified 32 genes which were methylated in SSAs compared with normal mucosa, and performed immunohistochemistry for the six most promising candidates. *HDHD3*, which was also identified in our study (see Table 2), was one of the candidates. Parker et al. [38] compared SSAs with normal mucosa using the SeqCap Epi CpGiant sequencing platform, followed by RNA sequencing. They were able to construct a six-gene methylation panel which separated SSAs from normal mucosa.

The genes identified in the studies above are useful in understanding the earliest steps in SSA formation. However, the clinical challenge is in identifying markers which separate advanced SSAs from ordinary SSAs, as the former is capable of rapid progression to CRC. In this vein, Andrew et al. [39] used the HumanMethylation450 array to identify 15 CpG probes methylated in CIMP-high CRCs as well as SSAs, and validated the results in several public datasets. Although these probes were all located within islands they were not restricted to promoters, and interestingly none corresponded to *MLH1*. In our study, methylation of these probes as assessed by the MethylationEPIC array did not differ significantly between SSAs and SSAD backgrounds (Additional file 6: Supplementary Table 5). This suggests the candidate probes identified by Andrew et al. are methylated early in SSA development and are not useful in separating SSAs from SSAD backgrounds.

Notably, these four studies also identified significant hypomethylation with lesion progression, whether it was from normal mucosa to SSA [36–38], or from SSA to CIMP-high CRC [39]. In our study, we have shown it also occurs from SSA to SSAD background, at an even higher frequency than hypermethylation. This indicates hypomethylation and hypermethylation are changes that accumulate gradually at all steps of SSA progression to CRC. Although the functional consequences of hypomethylation could not be explored via the methods employed in our study, it is known hypomethylation does not represent a bystander process or the simple mirror image of hypermethylation [40]. Global hypomethylation can induce gross chromosomal abnormalities [41], while hypomethylation of repetitive sequences such as long interspersed nuclear elements-1 (LINE-1) facilitates their insertion into regulatory regions and tumour-suppressor genes [42]. In contrast, direct activation of proto-oncogenes by hypomethylation appears to be an uncommon event [43].

Together, the four studies described above utilised small numbers of lesions and several analysis methods to identify differentially methylated genes in the development of non-dysplastic SSAs. The obtained data showed minimal overlap, and no constant methylation event was identified across all studies. Although this may have been due to methodology differences, it is also possible SSAs do not differentially methylate a set of predefined genes during its long dwell time prior to developing dysplasia. Notably, none of these studies identified *MLH1* as a candidate, consistent with the view that *MLH1* methylation represents an advanced alteration seen only in SSADs.

Since our methylation array data demonstrated only small fold changes (logFC range between −2.0 and + 2.1 for all significantly different probes), we examined genes with known methylation statuses, to ensure data consistency across methods. Because all SSAD backgrounds and SSAs were CIMP-high via MethyLight, the five CIMP genes were expected to be similarly methylated on the MethylationEPIC array across all lesions. Indeed, when promoter-associated probes corresponding to the five CIMP genes were considered, the majority showed no significant difference between SSAD backgrounds and SSAs (Additional file 2: Supplementary Figure 1). The six probes which did show a significant difference were all hypermethylated in SSAD backgrounds, consistent with the observation they represented more advanced SSAs. As a further means of validating our data, we also compared our methylation data with *BRAF*-mutant CIMP-high microsatellite unstable CRCs. These CRCs were the expected end result if the MLH1-deficient SSADs in our study were allowed to progress. Of the assessable probes, 94.6% were concordant between SSAD background and CRC, suggesting the methylation changes which occur from SSA to SSAD background persist into CRC.

To identify potential tumour-suppressor genes silenced in the progression of SSA to SSAD background, we limited analyses to hypermethylated, island-located probes within the promoters of protein-coding genes. We then compared the results to expression data derived from *BRAF*-mutant CIMP-high microsatellite unstable CRCs, to further narrow down the candidates to genes which were downregulated by promoter hypermethylation.

Of the genes corresponding to the top probes, *ADAMTS19*, *PGR*, *MLH1*, *HIC1*, *EN1*, *OGDHL* and *LHX6* methylation have been described in CRCs, and *HDHD3* methylation has been described in SSAs. *ADAMTS19* methylation is associated with mucinous differentiation, *BRAF* mutation, microsatellite instability and metastasis [44]. *PGR* codes for the progesterone receptor. Because hormone replacement therapy reduces CRC risk [45], progesterone receptor likely functions as a tumour suppressor. This is supported by cell line [46] and murine [47] data. However, hormone replacement therapy does not significantly affect *PGR* methylation [48], and the protective effect of hormone replacement therapy may be due to oestrogen receptor rather than progesterone receptor function. *HIC1* has been investigated mainly as part of a CIMP panel [49], but its methylation is not associated with clinicopathologic features [50]. *EN1* methylation is also not associated with clinicopathologic features [51]. *OGDHL* is methylated and downregulated in a proportion of unselected CRC [52]. *LHX6* methylation is described in CRC [53], but its functional significance has not been investigated. *HDHD3* is methylated in SSAs, where decreased expression is confirmed by immunohistochemical staining [37].

*MLH1* warrants separate discussion as its role in SSA progression is well established. Previous studies have demonstrated *MLH1* methylation is detectable in otherwise unremarkable SSAs, ranging from 0.0 to 89.5% [13, 28, 31, 32, 54–65]. The cause for this large variation is uncertain but has been attributed to patient ethnicity [58], patient age [13, 60], lesion site [31, 55, 58] and primer choice [58, 66]. In SSADs, there is a very high concordance rate between *MLH1* methylation and immunohistochemical MLH1 loss in the dysplastic portion [7]. These prior studies have led to the view that *MLH1* methylation occurs late in SSA progression, where once a critical threshold is reached, MLH1 protein function is lost and there is concurrent development of dysplasia.

Our current data further clarify the role of *MLH1* in the progression of SSA to SSAD. Because all our SSAD backgrounds were associated with MLH1-deficient dysplasia, it was not surprising to identify *MLH1* methylation within this background. SSAD backgrounds were much more likely to be *MLH1* methylated via MethyLight compared with SSAs (62.9% vs. 0.0%), but this difference was enhanced by the requirement that all SSAs were *MLH1* unmethylated. Our results were different to a previous study of macrodissected SSADs, which found *MLH1* methylation did not differ significantly between ordinary SSAs and SSAD backgrounds [67]. This was likely due to their inclusion of both MLH1-deficient and MLH1-retained SSADs.

In our study, 52.5% of SSAD backgrounds showed MLH1 loss in isolated crypts (Fig. 1). However, it should be noted that because these foci represented less than 5% of the crypts in each histologic section, undersampling was possible and the true incidence would likely be higher if additional sections were taken. Compatible with our result, a recent study demonstrated focal MLH1 loss in 65.7% of SSAD backgrounds from MLH1-deficient SSADs [9]; furthermore, although we did not perform MLH1 immunohistochemistry in our SSAs, focal MLH1 loss was identified in 7.0% of randomly selected SSAs in the same study [9]. Focal MLH1 loss in SSAs and SSAD backgrounds had also been illustrated in other studies [32, 68–71], mainly as a pathologic curiosity.

The *MLH1* methylation model can be improved as follows. *MLH1* methylation occurs late in SSA progression, concentrated within promoter-associated CpG islands. This methylation is heterogeneous across the lesion, and different crypts accumulate methylation at varying rates, with some eventually losing protein function, reflected by MLH1 loss on immunohistochemistry. This explains the discordant methylation and immunohistochemistry results, because not all sections from a given SSA background will include the hypermethylated crypts. It is unknown how long these MLH1-deficient crypts can persist before development of dysplasia. However, as they are rarely seen in the absence of overt dysplasia, the rate of progression is likely rapid.

The main limitation of our study was lack of expression data from SSAD backgrounds and SSAs, which were required to identify the genes silenced by hypermethylation. Because SSAs are CRC precursors, the entire lesion should be submitted for pathologic analysis to exclude a microscopic malignant component, and obtaining fresh tissue for RNA or protein extraction is difficult. As a surrogate, we utilised expression data derived from *BRAF*-mutant CIMP-high microsatellite unstable CRC. However, this involved comparing methylation data derived from formalin-fixed paraffin-embedded SSAD backgrounds and SSAs, and expression data derived from fresh-frozen CRCs and normal mucosa. Differences in tissue type, DNA/RNA quality, platform choice and data analysis were confounding factors in this part of the study.

## Conclusion

We have shown significant methylation changes occur during SSA progression, well before CRC and even before development of histologic dysplasia. In addition to expected hypermethylation of promoter-associated CpG islands, there is also hypomethylation of shore, shelf and sea regions. These methylation changes likely persist into the resulting CRC. Furthermore, methylation is a heterogeneous process even within individual SSAs, where isolated MLH1-deficient crypts can occur in a MLH1-retained background, in the absence of histologically detectable dysplasia. This heterogeneity is likely a generalised phenomenon involving multiple genes in addition to *MLH1* and should be taken into account in future methylation-based studies. Furthermore, this result has implications in the development of clinical methylation panels, as these panels generate methylation data averaged across entire lesions, where small foci of significant methylation may be diluted by the background.

## Methods

**Fig. 3.**
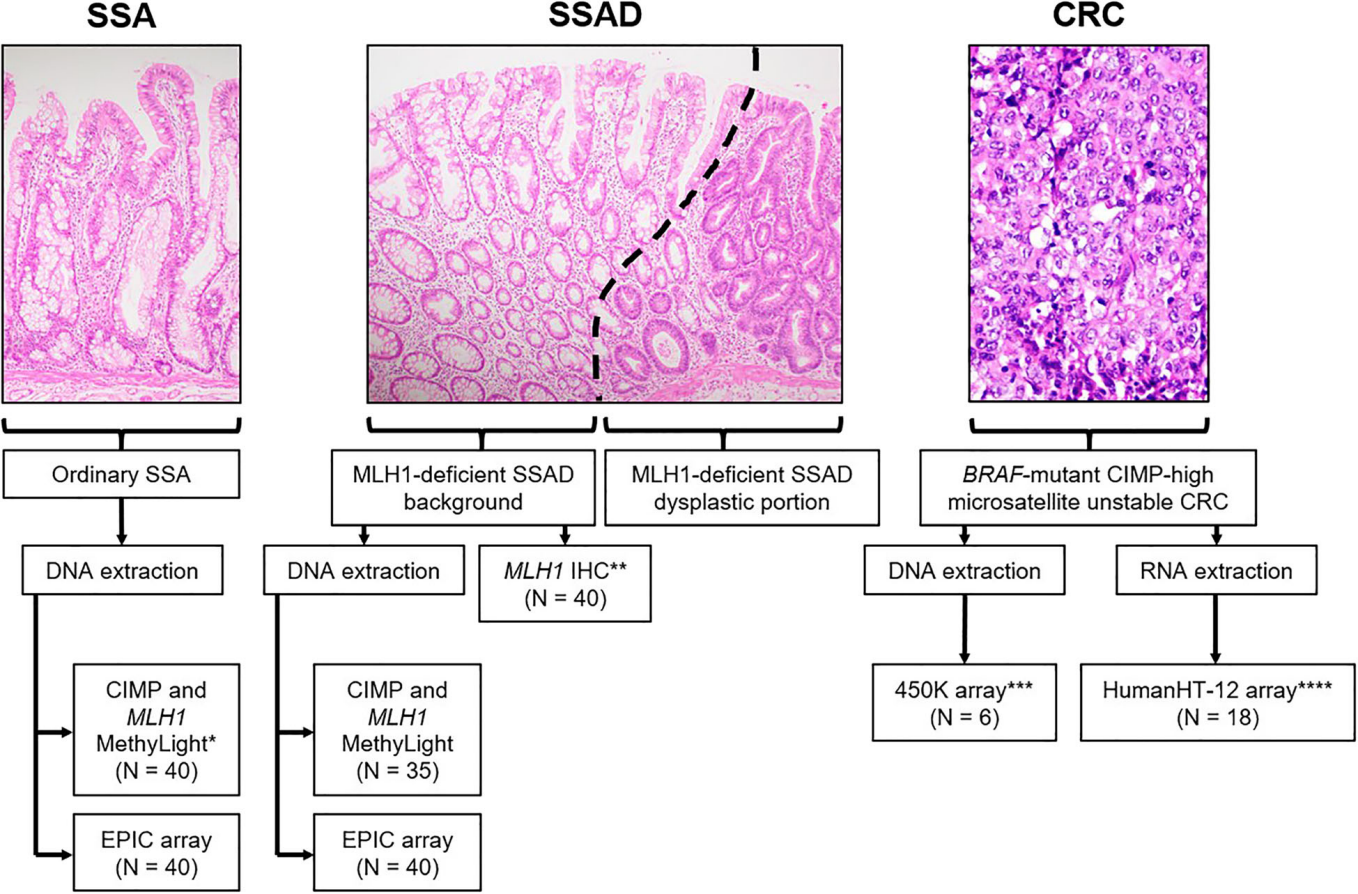
Experimental design. An SSA progresses to SSAD and then to *BRAF*-mutant CIMP-high microsatellite unstable CRC. Within SSADs, the SSAD background represents the most advanced SSA prior to dysplasia and should also demonstrate methylation differences from ordinary SSAs. Asterisks indicate data obtained from previous studies. *Data obtained from Liu et al. [13]; all SSAs were also required to be *MLH1* unmethylated via MethyLight for inclusion in the current study. **Data obtained from Liu et al. [10]. ***Data obtained from Dumenil et al. [18]. ****Data obtained from Fennell et al. [21]. CRC, colorectal cancer; SSAD, sessile serrated adenoma with dysplasia; SSA, sessile serrated adenoma; CIMP, CpG island methylator phenotype; EPIC, Illumina MethylationEPIC platform; 450K, Illumina HumanMethylation450 platform; HumanHT-12, Illumina HumanHT-12 v3 Expression platform; IHC, immunohistochemistry.

A schematic of our experimental design is shown in Fig. 3.

### Case selection

A total of 40 SSADs were sourced from Envoi Specialist Pathologists, a gastrointestinal pathology practice in Brisbane, Australia. For each SSAD, haematoxylin and eosin (H&E)-stained slides were retrieved and reviewed by two gastrointestinal pathologists (C. L. and M. L. B.) to confirm the SSAD fulfilled the World Health Organization diagnostic criteria. Furthermore, the SSADs were required to (1) have sufficient SSAD background component for macrodissection (at least 3 mm) and (2) demonstrate loss of MLH1 staining on immunohistochemistry in their dysplastic component. The second criterion was introduced because MLH1-deficient SSADs were more common than MLH1-proficient SSADs, and it restricted analysis to a homogeneous group. Patient demographic data and lesion site (proximal or distal colon) were obtained from the pathology request form. Of these 40 SSADs, 33 had been reported in two previous studies [7, 10].

### DNA extraction

For each SSAD, the corresponding paraffin block was retrieved. Ten 10-μm unstained sections were cut onto uncharged slides, followed immediately by a single 4 μm H&E section. This additional H&E section was used to confirm the SSAD background remained in the intervening unstained sections. The unstained sections were deparaffinised, and the SSAD background was macrodissected using a sterile needle and the original H&E section as a guide; to avoid contamination by the dysplastic focus, a thin rim of SSAD background immediately adjacent to the dysplastic focus was not included in the macrodissection. Lesional mucosa content was at least 90% for all cases. DNA was extracted via Chelex as previously described [72].

### CIMP and *MLH1* methylation

After prioritisation of SSAD background DNA for methylation microarray, there was sufficient sample remaining to assess CIMP and *MLH1* methylation status by MethyLight in 35 of 40 SSAD backgrounds. This was performed as previously described [73]. CIMP used the five markers of Weisenberger et al. [16] (*NEUROG1*, *SOCS1*, *CACNA1G*, *IGF2* and *RUNX3*), where CIMP-high was defined as three or more markers methylated.

### Control selection

For each SSAD, an age-, gender-, site- and CIMP status-matched ordinary SSA was selected as control. The SSAs were also required to be *MLH1* unmethylated. All 40 selected SSAs had been included in a previous study [13], which used identical methods of DNA extraction and MethyLight analysis. Because SSADs are overwhelmingly proximal colonic and CIMP-high [7], lesions without a stated site of origin (three SSADs) were matched with a proximal SSA, and lesions with insufficient DNA for CIMP (five SSADs) were matched with a CIMP-high SSA.

With this method of control selection, the study utilised two very closely related, but distinct, groups. The SSAD backgrounds represented the most advanced SSAs prior to development of MLH1-deficient dysplasia, while the matched SSAs represented the highly methylated lesions typically seen in an older population [13]. The *MLH1*-unmethylated requirement in the SSAs enhanced the difference between the groups, which was intended to exclude undersampled SSADs not present in the planes of the section. Any differences between these two groups would therefore represent late events in SSA progression, occurring just prior to MLH1 loss.

### Methylation microarray

For all SSAD backgrounds and SSAs, 1.0 μg of extracted DNA was submitted to Macrogen, Inc. (Seoul, South Korea) for methylation microarray. DNA quality control used the Infinium FFPE QC Kit (Illumina, San Diego, USA), DNA restoration used the Infinium HD FFPE DNA Restore Kit (Illumina, San Diego, USA), bisulfite conversion used the EZ-96 DNA Methylation Kit (Zymo Research, Irvine, USA) and methylation microarray used the Infinium MethylationEPIC BeadChip Kit (Illumina, San Diego, USA).

### Immunohistochemistry

MLH1 immunohistochemistry had been previously performed on all SSADs as part of a previous study [10].

### Methylation array data analysis

MethylationEPIC array data were imported into the R environment using *minfi* (v1.28.3) [74]. Probes were annotated using the mapping by Zhou et al. [75], and probes that mapped to single nucleotide polymorphisms or sex chromosomes were not included in downstream analyses. Data were normalised using the functional normalisation method [76]. We masked *β* values for individual positions with detection *P* ≥ 0.05 and discarded any probes with detection *P* ≥ 0.05 in more than 50% of samples. Differential methylation analyses were performed on the M-transformed *β* values using the Limma package (v3.38.3) [77]. *P* values were adjusted to account for errors in multiple testing using the Benjamini-Hochberg false discovery rate method [78].

### Comparison with CRC data

We compared our global SSAD background and SSA methylation data with CRC methylation data from a previous study [18], which included six *BRAF*-mutant CIMP-high microsatellite unstable CRCs and also utilised DNA extracted from formalin-fixed, paraffin-embedded tissue. Because an earlier version of the methylation array was used (HumanMethylation450 instead of MethylationEPIC), we limited the comparison to probes present on both platforms. Raw data were normalised using functional normalisation and quality control filtering as above.

Because promoter methylation does not always lead to transcriptional silencing [19, 20], we investigated whether significantly methylated genes in SSAD backgrounds correlated with reduced expression in CRCs. To this end, we examined differential gene expression between 18 *BRAF*-mutant CIMP-high microsatellite unstable CRCs and 32 normal colonic mucosa samples from a publicly accessible gene expression dataset (Fennell et al. [21], ArrayExpress E-MTAB-7036). Raw data were imported into the R environment, quantile normalised and background corrected. Where multiple probes mapped to a single gene, we computed the median to assess gene-level expression. Differential gene expression was performed using the Limma package (v3.38.3), and *P* values were corrected for false discovery using the Benjamini-Hochberg false discovery rate method as above.

### Statistical analysis

Continuous variables were analysed using an unpaired *t*-test. Categorical variables were analysed using a chi-squared test. A *P* value of < 0.05 was regarded as significant.

### Availability of methylation array data

The dataset supporting the conclusions of this article is available in the ArrayExpress repository (E-MTAB-7854).

## Additional files

Additional file 1: Supplementary **Table 1.** Detailed CIMP and *MLH1* MethyLight results. (DOCX 13 kb)

Additional file 2: Supplementary** Figure 1.** Methylation of CIMP gene and *MLH1* promoters as assessed by the MethylationEPIC array. Each data point represents a promoter-associated probe, plotted as the *β* value difference between SSAD backgrounds and SSAs (*β*_SSAD backgrounds_ − *β*_SSAs_). A positive value indicates hypermethylation in the SSAD background, and a negative value indicates hypomethylation in the SSAD background. Significantly different probes are highlighted in red. TSS, transcription start site. (DOCX 40 kb)

Additional file 3: Supplementary **Table 2.** Correlation between MethyLight *MLH1* methylation and immunohistochemical MLH1 loss in SSAD backgrounds. (DOCX 12 kb)

Additional file 4: Supplementary **Table 3.** Genes with at least one hypermethylated promoter island probe. (XLSX 670 kb)

Additional file 5: Supplementary **Table 4.** Differentially expressed genes in *BRAF*-mutant microsatellite unstable CRCs, as compared with our list of hypermethylated gene promoter-associated probes. (XLSX 42 kb)

Additional file 6: Supplementary **Table 5.** The probes identified by Andrew et al. [39], as assessed on the MethylationEPIC array in our study. (XLSX 11 kb)

## Data Availability

The datasets generated and analysed during the current study are available in the ArrayExpress repository (E-MTAB-7854).
