# Supplementary material for: DNA methylation changes that precede onset of dysplasia in advanced sessile serrated adenomas
Source: Clin Epigenetics. 2019 Jun 14;11:90. doi: 10.1186/s13148-019-0691-4 (PMC6570920; doi:10.1186/s13148-019-0691-4)
Supplement: Supplementary file 1 — Supplementary Table 1. Detailed CIMP and MLH1 MethyLight results. (DOCX 13 kb) [file 13148_2019_691_MOESM1_ESM.docx]

**Supplementary Table 1. Detailed CIMP and *MLH1* MethyLight results.**

|  |  | SSAD background (N = 35) | SSA (N = 40) | P value |
| --- | --- | --- | --- | --- |
| CIMP |  |  |  |  |
|  | CIMP-high | 35 | 40 | N. S. |
|  | Non-CIMP-high | 0 | 0 |  |
| *NEUROG1* |  |  |  |  |
|  | Methylated | 35 | 39 | N. S. |
|  | Unmethylated | 0 | 1 |  |
| *SOCS1* |  |  |  |  |
|  | Methylated | 28 | 20 | < 0.01 |
|  | Unmethylated | 7 | 20 |  |
| *CACNA1G* |  |  |  |  |
|  | Methylated | 35 | 35 | N. S. |
|  | Unmethylated | 0 | 5 |  |
| *IGF2* |  |  |  |  |
|  | Methylated | 35 | 37 | N. S. |
|  | Unmethylated | 0 | 3 |  |
| *RUNX3* |  |  |  |  |
|  | Methylated | 35 | 35 | N. S. |
|  | Unmethylated | 0 | 5 |  |
| *MLH1* |  |  |  |  |
|  | Methylated | 22 | 0 | < 0.01 |
|  | Unmethylated | 13 | 40 |  |

SSAD, sessile serrated adenoma with dysplasia; SSA, sessile serrated adenoma; CIMP, CpG island methylator phenotype; N. S., not significant.
