# Supplementary material for: DNA methylation changes that precede onset of dysplasia in advanced sessile serrated adenomas
Source: Clin Epigenetics. 2019 Jun 14;11:90. doi: 10.1186/s13148-019-0691-4 (PMC6570920; doi:10.1186/s13148-019-0691-4)
Supplement: Supplementary file 2 — Supplementary Figure 1. Methylation of CIMP gene and MLH1 promoters as assessed by the MethylationEPIC array. Each data point represents a promoter-associated probe, plotted as the β value difference between SSAD backgrounds and SSAs (βSSAD backgrounds − βSSAs). A positive value indicates hypermethylation in the SSAD background, and a negative value indicates hypomethylation in the SSAD background. Significantly different probes are highlighted in red. TSS, transcription start site. (DOCX 40 kb) [file 13148_2019_691_MOESM2_ESM.docx]

|  |
| --- |
| 1A |
|  |
| 1B |
|  |
| 1C |
|  |
| 1D |
|  |
| 1E |
|  |
| 1F |

**Supplementary Figure 1. Methylation of CIMP gene and *MLH1* promoters.** For each graph, a positive β value difference indicates hypermethylation in SSAD background, and a negative β value difference indicates hypomethylation in SSAD background. Significantly different probes are highlighted in red. TSS, transcription start site.
