# Supplementary material for: DNA methylation changes that precede onset of dysplasia in advanced sessile serrated adenomas
Source: Clin Epigenetics. 2019 Jun 14;11:90. doi: 10.1186/s13148-019-0691-4 (PMC6570920; doi:10.1186/s13148-019-0691-4)
Supplement: Supplementary file 3 — Supplementary Table 2. Correlation between MethyLight MLH1 methylation and immunohistochemical MLH1 loss in SSAD backgrounds. (DOCX 12 kb) [file 13148_2019_691_MOESM3_ESM.docx]

**Supplementary Table 2. Correlation between MethyLight *MLH1* methylation and immunohistochemical MLH1 loss in SSAD backgrounds.**

|  | |  | MLH1 loss | No MLH1 loss | P value |
| --- | --- | --- | --- | --- | --- |
| Using ≥ 10% PMR cut off | | |  |  |  |
|  | *MLH1* methylated | | 14 | 8 | N. S. |
|  | *MLH1* unmethylated | | 5 | 8 |  |
| Using > 0% PMR cut off | | |  |  |  |
|  | *MLH1* methylated | | 17 | 10 | N. S. |
|  | *MLH1* unmethylated | | 2 | 6 |  |

SSAD, sessile serrated adenoma with dysplasia; PMR, percentage of methylated reference; N. S., not significant.
